# Two-dimensional speckle tracking echocardiography help identify breast cancer therapeutics–related cardiac dysfunction

**DOI:** 10.1186/s12872-022-03007-8

**Published:** 2022-12-15

**Authors:** Wei Liu, Wei Li, Hairu Li, Ziyao Li, Peng Zhao, Zihong Guo, Cong Liu, Litao Sun, Zhenzhen Wang

**Affiliations:** 1 Department of Ultrasound Medicine, Heart Center, Affiliated People’s Hospital, Zhejiang Provincial People’s Hospital, Hangzhou Medical College, 158 Shangtang Rd., Hangzhou, China; 2 Department of Ultrasound Medicine, Second Affiliated Hospital of Harbin Medical University, Harbin, China

**Keywords:** Two-dimensional speckle tracking echocardiography, Breast cancer, Neoadjuvant chemotherapy, Left ventricular systolic function, Cancer therapeutics–related cardiac dysfunction

## Abstract

**Background:**

Cancer therapeutics–related cardiac dysfunction (CTRCD) from different chemotherapy strategies are underdetermined by echocardiography. As an imaging marker of subclinical cardiac dysfunction, two-dimensional speckle tracking echocardiography (2D-STE) may assist in identifying the impact patterns of different CTRCD.

**Methods:**

A total of 67 consecutive patients with invasive ductal breast carcinoma who will undertake neoadjuvant chemotherapy were enrolled and grouped according to their different chemotherapy regimens based on their biopsy results. Group A included 34 patients who received anthracycline without trastuzumab, whereas Group B had 33 patients who received trastuzumab without anthracycline. Echocardiography was performed at three time-points, i.e., baseline (T0), cycle-2 (T2), and cycle-4 (T4) of chemotherapy. Conventional echocardiographic measurements and 2D-STE strain values, and myocardial work (MW) parameters, were compared between different groups at different time-points.

**Results:**

The mean age had no statistical difference between the two groups. E/e′ was the only conventional echocardiographic parameter that had variation in group A (*P* < 0.05). Compared with baseline, GLS in group A decreased at T2, and GCS decreased at T4 (*P* < 0.05). GLS and GCS in group B both decreased at T4 (*P* < 0.05). More patients in group A had a more than 15% fall of baseline GLS rather than GCS at T2 (*P* < 0.05), however, there was no difference of either GLS or GCS decline rate at T4 between the two groups. All the MW parameters in group A had variations overtime, whereas only GCW in group B (*P* < 0.05).

**Conclusion:**

Early subclinical myocardial dysfunction can be identified by 2D-STE in breast cancer patients with chemotherapy, and GLS provides profound value in demonstrating the temporal changes in early myocardial damage induced by anthracycline. LV contractility injury in patients with trastuzumab may be mild at first but increases in severity with exposure time as early as cycle-4. Awareness of these differences may help to stratify the prevention of late cardiovascular events caused by different CTRCDs. In addition, GCW may be the most sensitive myocardial work parameter of CTRCD.

## Background

Anthracycline and trastuzumab have been widely used in preoperative neoadjuvant chemotherapy for breast cancer patients as classic agents [1]. However, the cardiotoxicity of these drugs may significantly increase the risk of cancer therapeutics–related cardiac dysfunction (CTRCD) and heart failure [2, 3]. Therefore, early diagnosis of CTRCD and risk stratification is somehow mandatory. The CTRCD derived from the two types of agents have implied a variant form of myocardial dysfunction owing to their different and complex mechanisms [4–6]. The myocardial impact caused by the anthracycline occurs from the earliest administration of the drug in a cumulative dose-dependent fashion and was categorized as type-I CTRCD [3]. On the contrary, some agents do not make severe damage in a dose-dependent manner and have been considered reversible. The typical example is trastuzumab which has been documented as type-II CTRCD agent, primarily regarding the HER2-positive breast cancer [7]. Cardiac dysfunction resulting from exposure to chemotherapy was first recognized with the widespread application of anthracyclines in 1960s [3]. Echocardiography is the preferred method for the evaluation of cardiac involvement of cancer patients before, during and after chemotherapy. And monitoring of left ventricular (LV) ejection fraction (LVEF) has evolved as a widely used strategy to detect anthracycline-induced LV dysfunction for decades [8]. However, LVEF may not be sufficient to detect small changes in LV contractility, and an overt decreased LVEF maybe too late for prevention of myocardial dysfunction or heart failure [3, 9]. Given the myocardium have considerable reserves, sensitive imaging approach is urgently needed.

Two-dimensional speckle tracking echocardiography (2D-STE) provides biomechanical parameters that may represent LV myocardial deformation (strain), including mainly global longitudinal strain (GLS) and the global circumferential strain (GCS) [10, 11]. Strain is considered more sensitive in early identifying subclinical myocardial dysfunction in patients with preserved ejection fraction, compared with conventional echocardiography [12, 13]. Emerging evidence shows that the 2D-STE is a good surrogate in assessing the myocardial deterioration derived from chemotherapy, and a 15% absolute fall in LV-GLS of the baseline during follow-up represents the presence of myocardial toxicity [3, 14, 15]. Given that strain measurements also have important load dependency [16], we also introduce myocardial work (MW) parameters to provide loading-independent evaluation of myocardial performance. Here we aim to compare the subclinical myocardial impairment of different chemotherapy agents in breast cancer patients with preserved ejection fraction by 2D-STE. Not only to test if 2D-STE could early differentiate type-I CTRCD from type-II CTRCD, but also provide evidence that 2D-STE could help in early risk stratification of different CTRCD.

## Methods

### Study population

**Fig. 1:**
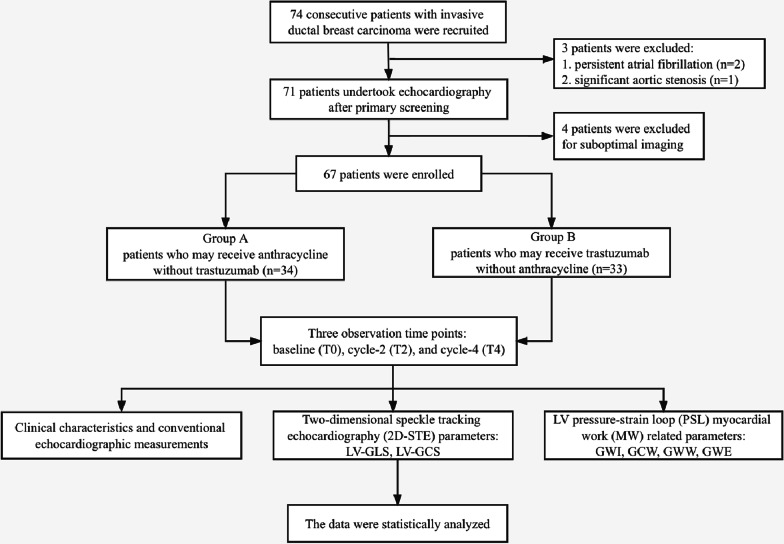
Flowchart of the patient enrollment

In this study we recruited breast cancer patients with pathologically confirmed invasive breast carcinoma by needle biopsy, between January 2020 and May 2021, in the breast surgery department of the Second Affiliated Hospital of Harbin Medical University. They were stratified into two groups according to their different neoadjuvant chemotherapy regimens as group A (anthracycline group) and group B (trastuzumab group). The patients should be newly diagnosed and untreated, have no history of other malignancy, LVEF > 50% by transthoracic echocardiography, and without abnormality in ECG or laboratory test. All patients had three comprehensive echocardiography and 2D-STE evaluation at baseline (before chemotherapy, T0), cycle-2 (T2), and cycle-4 (T4) of chemotherapy. None of the enrolled patients received radiotherapy before or during chemotherapy. At baseline and follow-ups, physicians who performed the echocardiography were blinded to patients’ pathological results and chemotherapy regimen. Finally, we enrolled 67 patients (34 in group A, and 33 in group B) who had comprehensive data in this study (see flowchart, Fig. 1). The study was approved by the ethics committee of the Second Affiliated Hospital of Harbin Medical University, and all patients signed written informed consents.

### Echocardiography

All the images were acquired on GE Vivid E9 ultrasound system with a M5S probe (1.5–4.5 MHz). Patients were placed in the left lateral decubitus position and a synchronized-lead ECG was connected. Conventional echocardiography and 2D-STE strain and MW assessment were performed by one advanced sonographer (Z Li) according to the ASE recommendations. Based on the cardio-oncology echocardiography protocol [3], 2D strain imaging was acquired ≥ 3 cardiac cycles from apical three-, four-, and two-chamber views with a framerate more than 40 frames/s. LV segmental strains and global longitudinal strain (GLS) were calculated. Additionally, LV global circumferential strain (GCS) was also evaluated from three LV short‐axis views (basal-, papillary-, and apical levels). MW is derived from pressure strain loop (PSL) analysis and provide parameters such as global work index (GWI), global constructive work (GCW), global work waste (GWW), and global work efficiency (GWE). The raw DICOM data was imported into the Echo PAC workstation and the strain as well as MW measurements were conducted manually according to the 2D-STE protocol, and all strains were considered as absolute values for statistical analysis.

#### Statistical analysis

Continuous variables were summarized as means ± standard deviations, and category variables were recorded as number with percentage. We used the student t-test to compare the continuous variables between the two groups. Parameters in each group at different time-points were compared by using one-way analysis of variance. The chi-square tests were introduced to compare category variables. We used SPSS version 25.0 (IBM Corporation, Armonk, NY) statistical software. A two-tailed *P* value less than 0.05 was considered statistically significant.

## Results

### Patient baseline characteristics

The clinical characteristics at baseline of group A and B are shown in Table 1. Patient age in group A was 48 ± 6 years-old and 51 ± 6 years-old in group B (*P* = 0.07). There were no statistical differences in age, body mass index, blood pressure, heart rate, cardiovascular disease history and medication between the two groups.

**Table 1:** Characteristics of the Study Population

Characteristics	Group A (N = 34)	Group B (N = 33)	*P*
Age (years)	48 ± 6	51 ± 6	0.07
BMI (kg/m^2^)	22.61 ± 2.51	23.52 ± 2.81	0.16
SBP (mm Hg)	119.00 ± 11.54	120.84 ± 13.38	0.55
DBP (mm Hg)	79.00 ± 7.38	81.16 ± 8.30	0.26
h (bpm)	73.65 ± 4.29	73.84 ± 7.06	0.89
Cardiovascular history*, n (%)			
Hypertension	9 (26.5%)	11 (33.3%)	0.53
Diabetes mellitus	5 (14.7%)	6 (18.2%)	0.70
Medications, n (%)			
Statin	7 (20.6%)	4 (12.1%)	0.51
Beta-blocker	4 (11.4%)	6 (16.7%)	0.76
ACEI or ARB	5 (14.7%)	7 (21.2%)	0.48
Calcium channel blocker	7 (20.6%)	8 (24.2%)	0.72

*Pre-existing known cardiovascular diseases other than hypertension and diabetes mellitus have been excluded before patient recruitment

*BMI* Body mass index, *SBP* Systolic blood pressure, *DBP* Diastolic blood pressure, *HR* Heart rate, *ACEI* Angiotensin converting enzyme inhibitor, *ARB* Angiotensin II receptor blocker

### Conventional echocardiography

Conventional echocardiography parameters at three different time-points are shown in Table 2. LVEF was found preserved in both groups at each time-point. Among all echocardiographic variables, only E/e′ had variation within group A (*P* = 0.04).

**Table 2:** Differences of conventional echocardiography and 2D-STE parameters in each group of three time-points

Group	Time- point	LAV (ml)	LAVI (ml/m^2^)	IVS (mm)	LVPW (mm)	LVEDD (mm)	LVESD (mm)	LV-mass (g)	Simpson- LVEF(%)	M- LVEF(%)	E/A	E/e′	TAPSE (cm)
A	T0	32.91 ± 5.92	20.35 ± 2.92	9.17 ± 0.81	9.49 ± 0.81	43.58 ± 2.07	25.50 ± 2.28	110.86 ± 12.19	66.78 ± 4.17	66.03 ± 2.48	1.06 ± 0.23	7.75 ± 1.76	2.18 ± 0.30
	T2	32.18 ± 5.01	20.07 ± 2.64	9.35 ± 0.74	9.49 ± 0.80	43.87 ± 3.03	26.24 ± 2.25	112.82 ± 8.80	67.36 ± 4.29	65.95 ± 3.07	1.07 ± 0.31	7.49 ± 1.75	2.13 ± 0.29
	T4	33.19 ± 5.23	21.04 ± 3.21	9.17 ± 0.79	9.30 ± 0.79	43.23 ± 2.79	25.79 ± 2.51	113.16 ± 10.74	66.30 ± 3.13	65.17 ± 2.17	1.00 ± 0.18	6.83 ± 1.60	2.15 ± 0.28
Group A	P^a^	0.41	0.16	0.36	0.29	0.41	0.42	0.48	0.80	0.25	0.06	0.04	0.46
B	T0	33.93 ± 5.44	20.56 ± 3.70	9.18 ± 0.80	9.51 ± 0.80	43.88 ± 3.02	25.05 ± 2.45	110.64 ± 15.31	66.14 ± 2.82	65.92 ± 3.40	1.03 ± 0.27	8.33 ± 1.38	2.19 ± 0.22
	T2	33.23 ± 5.35	20.11 ± 3.09	9.39 ± 0.83	9.50 ± 0.68	42.97 ± 2.51	25.49 ± 2.14	114.49 ± 15.59	66.03 ± 3.51	65.65 ± 2.75	0.97 ± 0.31	8.69 ± 1.44	2.15 ± 0.23
	T4	33.68 ± 6.12	21.16 ± 3.21	9.17 ± 0.61	9.40 ± 0.71	43.85 ± 3.10	25.40 ± 2.27	114.78 ± 12.01	65.08 ± 2.69	64.95 ± 1.67	0.99 ± 0.22	8.86 ± 1.75	2.10 ± 0.17
Group B	P^a^	0.75	0.23	0.24	0.70	0.24	0.66	0.20	0.19	0.45	0.60	0.68	0.08
Time	P^b^	0.40	0.06	0.09	0.52	0.71	0.66	0.09	0.72	0.13	0.12	0.56	0.12
Time*Group	P^c^	0.86	0.96	0.97	0.50	0.13	0.54	0.70	0.94	0.95	0.17	0.13	0.43

P^a^, P for each group among different time points; P^b^, P between groups at each time point; P^c^, P for interaction between group and time point; T0, Baseline; T2, Cycle 2 of chemotherapy, T4, Cycle 4 of chemotherapy; LAV, Left atrial volume; LAVI, Left atrial volume index; IVS, Interventricular septal thickness; LVPW, Left ventricular posterior wall thickness; LVEDD, Left ventricular end-diastolic diameter; LAESD, Left ventricular end‐systolic diameter; LVEF, Left ventricular ejection fraction; TAPSE, Tricuspid valve systolic displacement; E, Early peak mitral diastolic velocity; A, Late peak mitral diastolic velocity; e′, Early diastolic mitral annulus velocity

#### Two-dimensional speckle tracking echocardiography (2D-STE)

**Fig. 2:**
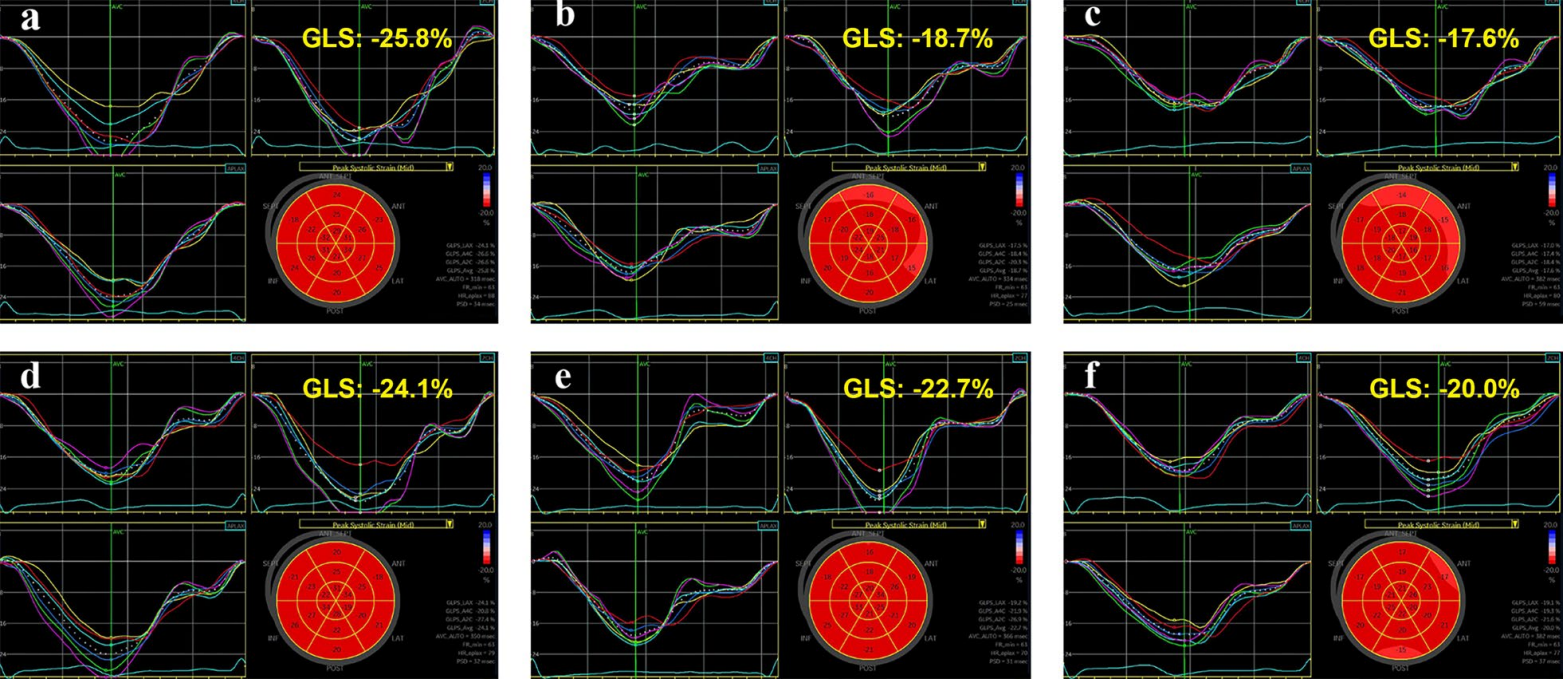
Speckle-tracking echocardiographic images illustrate longitudinal strain bull’s eye plot of baseline (**a**, **d**), cycle-2 (**b**, **e**) and cycle-4 (**c**, **f**) in patients undertook anthracycline (top panel), and trastuzumab (bottom panel), respectively. Each LV segment has a numeric and color-coded strain value. The normal color-code should be red, and the color-code of impaired segment may be different

**Fig. 3:**
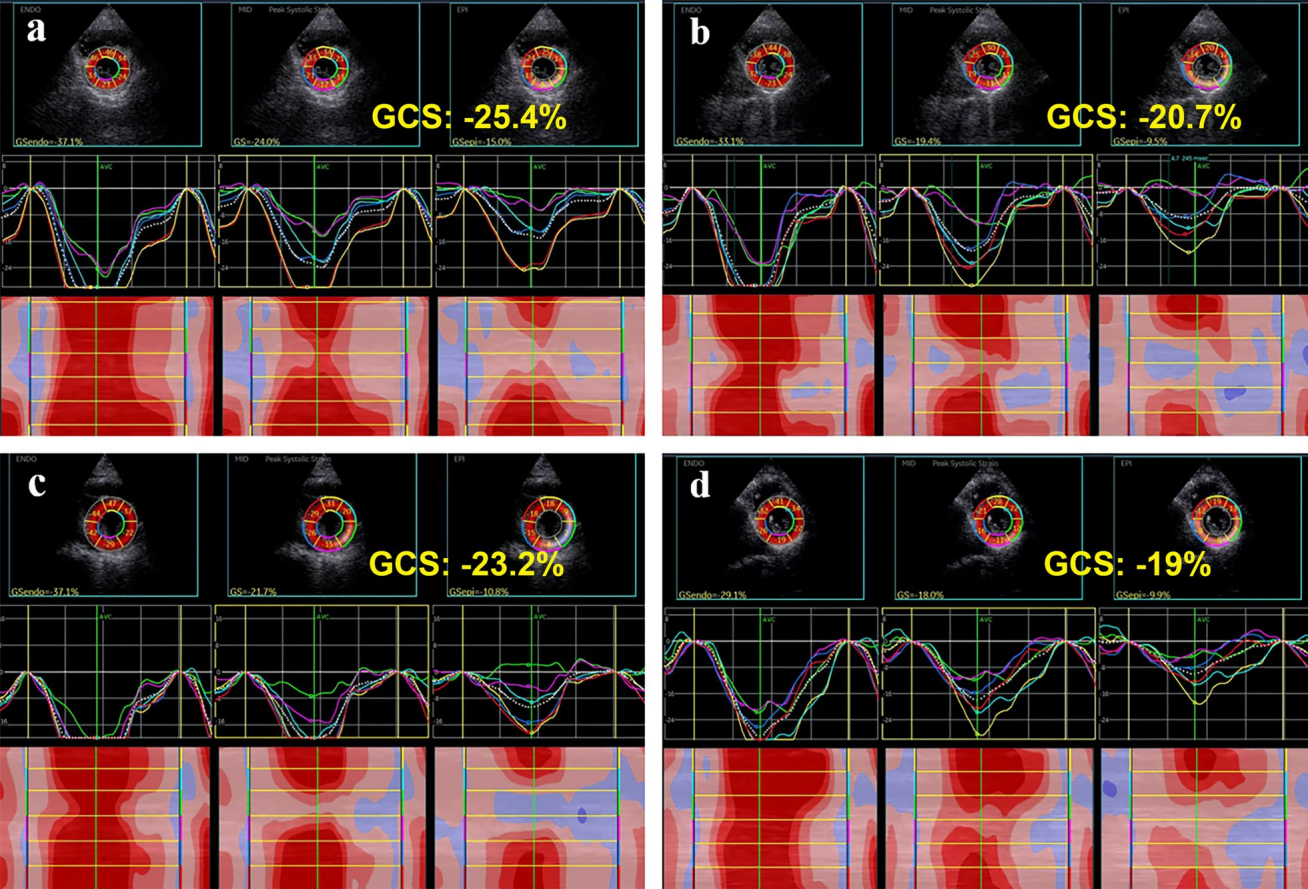
Speckle-tracking echocardiographic images illustrate LV-GCS in the parasternal short-axis views at the papillary muscle level of baseline (**a**, **c**) and cycle-4 (**b**, **d**) in two groups

**Fig. 4:**
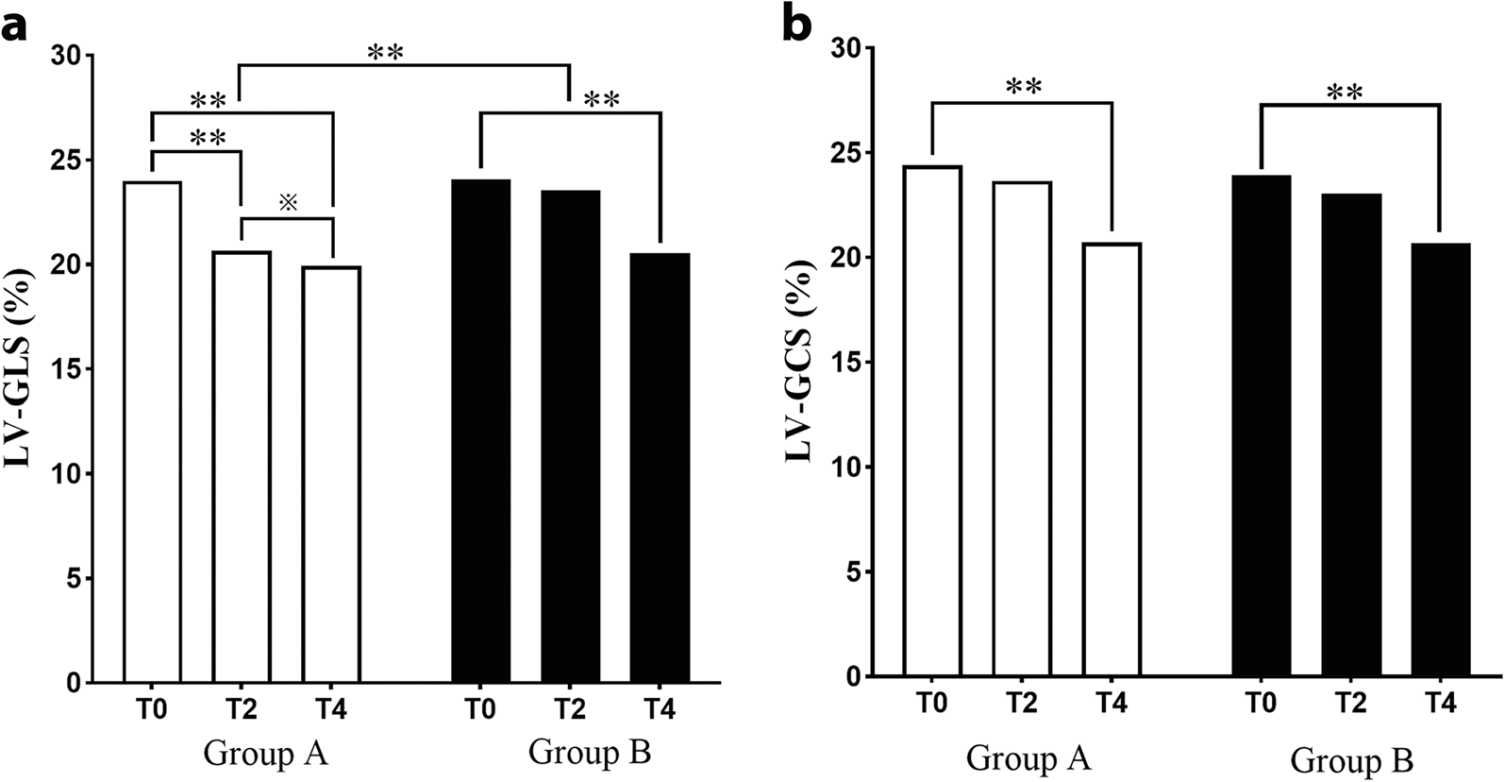
Histogram showing the differences of LV-GLS and LV-GCS between the two groups at different timepoints. **P < 0.005; ^※^*P* < 0.05. LV-GLS, Left ventricular global longitudinal strain; LV-GCS, Left ventricular global circumferential strain; T0, Baseline; T2, Cycle 2 of chemotherapy; T4, Cycle 4 of chemotherapy

Parameters of 2D-STE are presented in Table 3, and Figs. 2 and 3 demonstrate the bull’s eye plot regarding LV myocardial strains in two groups. Compared with baseline (T0), LV-GLS of cycle-2 (T2) and cycle-4 (T4) showed a timing decline pattern in group A (*P* < 0.005). Whereas in group B, the decrement was significant only at T4 (*P* < 0.005). In terms of LV-GCS, they both significantly reduced at T4 in each group (*P* < 0.005). In addition, the interaction analysis of strain values between group and different time-points was found statistically significant in GLS (*P* = 0.036), but not in GCS (Fig. 4).

**Table 3:** One-way ANOVA analysis of 2D-STE parameters in each group at different time-points

Group	Time-point	LV-GLS (%)	LV-GCS (%)
A	T0	24.00 ± 0.74	24.39 ± 1.45
	T2	20.66 ± 1.21**	23.65 ± 1.26
	T4	19.93 ± 0.78**^※^	20.71 ± 1.24**
B	T0	24.07 ± 0.98	23.91 ± 1.43
	T2	23.54 ± 0.88	23.03 ± 1.57
	T4	20.55 ± 1.58**	20.68 ± 1.58**
Time	P^a^	< 0.001	< 0.001
Time*Group	P^b^	0.036	0.119

Strain measurements are shown in absolute values. P^a^, P for each group at different time points; P^b^, P for interaction between group and time point; T0, Baseline; T2, Cycle 2 of chemotherapy, T4, Cycle 4 of chemotherapy; LV-GLS, LV global longitudinal strain; LV-GCS, LV global circumferential strain

Compared with T0, **P < 0.005

Compared with T2, ^※^*P* < 0.05

Given that an absolute fall in strain value of more than 15% of baseline during follow-up may represent myocardial toxicity, a chi-square test was used to compare the decline rate between two groups at two follow-up time-points (Table 4). Compared with group B, more patients in group A showed a decrement in LV-GLS of over 15% from baseline as early as T2 (*P* = 0.004), however, the decline rate in LV-GLS was found without significant difference at T4. Moreover, there was no difference regarding the LV-GCS decreasing rate between the two groups at either T2 or T4.

**Table 4:** Comparison of the decline rate of strain values (more than 15% absolute fall from baseline measurements) between the two groups at different time points

Group	N	LV-GLS (%)				LV-GCS (%)			
		T2, n (%)	*P*	T4, n (%)	*P*	T2, n (%)	*P*	T4, n (%)	*P*
A	34	17 (50.0%)	0.014	29 (85.3%)	0.126	8 (23.5%)	0.590	23 (67.6%)	0.730
B	33	7 (18.2%)		23 (69.7%)		6 (18.2%)		21 (63.6%)	

T0, Baseline; T2, Cycle 2 of chemotherapy, T4, Cycle 4 of chemotherapy; LV-GLS, LV global longitudinal strain; LV-GCS, LV global circumferential strain

Myocardial work (MW) parameters.

**Fig. 5:**
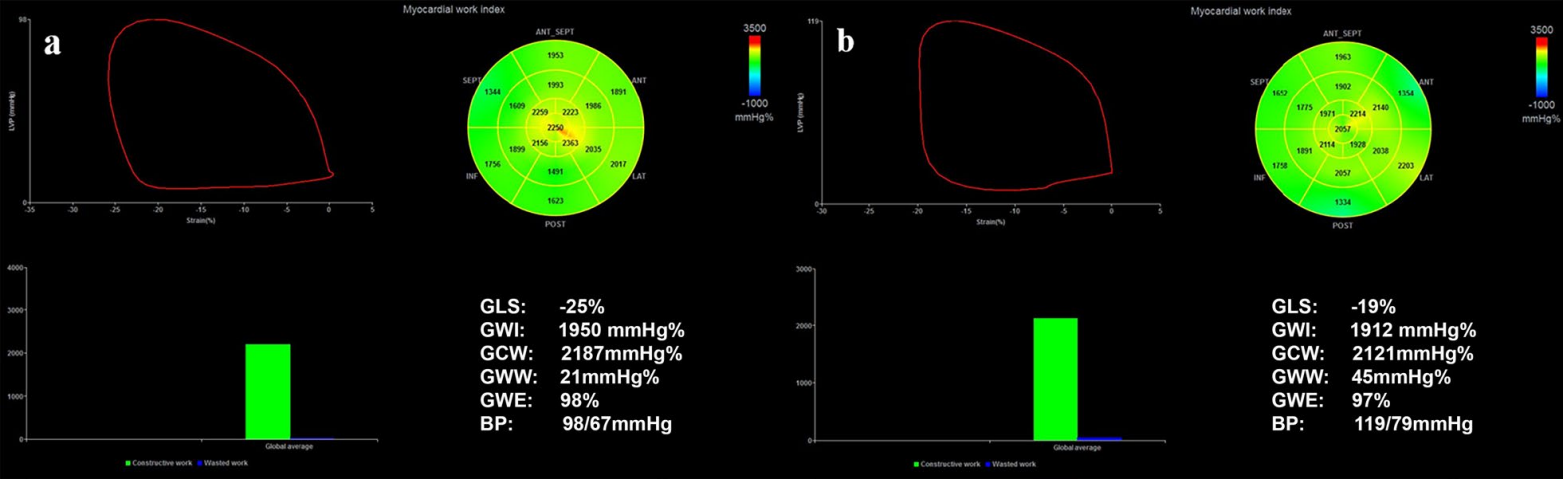
LV pressure-strain loop (PSL) myocardial work (MW) related parameters at baseline (**a**) and at cycle-4 (**b**) in anthracycline patients. The red curve in the upper left corner represents the PSL. The two bull’s-eye plots show the global work index (GWI), with negative work coded in blue, intermediate work in green, and positive work in red. The lower two green bars represent constructive myocardial work, whereas the blue ones represent wasted myocardial work

The MW parameters are shown in Table 5; Fig. 5. In group A, GWI, GCW and GWE decreased with time, whereas GWW increased gradually (*P* < 0.05). However, only the GCW in group B changed over time (*P* < 0.05). There had no interaction between group and different-time points regarding these MW parameters.

**Table 5:** Differences of myocardial work parameters in each group at three time-points

Group	Time-point	GWI (mmHg%)	GCW (mmHg%)	GWW (mmHg)	GWE (%)
A	T0	2050.59 ± 243.55	2256.59 ± 263.93	78.94 ± 14.68	96.29 ± 1.03
	T2	1986.26 ± 183.12	2167.97 ± 194.97	83.12 ± 14.89	96.18 ± 0.96
	T4	1923.68 ± 156.80	2066.85 ± 176.72	86.97 ± 10.96	95.85 ± 0.78
Group A	P^a^	0.016	< 0.001	0.013	0.027
B	T0	2042.82 ± 196.09	2233.39 ± 168.38	75.67 ± 16.74	96.61 ± 0.82
	T2	2015.21 ± 181.34	2161.85 ± 182.09	76.61 ± 13.92	96.39 ± 0.86
	T4	1959.39 ± 158.78	2101.73 ± 171.40	81.52 ± 13.43	96.24 ± 0.79
Group B	P^a^	0.129	0.001	0.094	0.082
Time	P^b^	0.001	< 0.001	0.002	0.003
Time*Group	P^c^	0.674	0.358	0.686	0.752

P^a^, P for each group among different time points; P^b^, P between groups at each time point; P^c^, P for interaction between group and time point; T0, Baseline; T2, Cycle 2 of chemotherapy; T4, Cycle 4 of chemotherapy; GWI, Global work index; GCW, Global constructive work; GWW, Global work waste; GWE, Global work efficiency

## Discussion

The two types of CTRCD are known to originate from different mechanisms, and type-I CTRCD is cumulatively dose dependent. Our study found the earliest temporal variation of myocardial deformation exists in patients undertake anthracycline but not in those with trastuzumab. To our knowledge, this is the first study to compare the temporal changes of early myocardial strain between the two types of CTRCD.

By noticing that only E/e′ had statistically difference within group A, our results indicated that conventional echocardiography parameters were not sufficient in early detecting either LV systolic or diastolic subclinical dysfunction. Although LVEF has been widely used as a remarkable measurement in monitoring the global systolic function during cancer treatment since 1960s, the patients may have preserved LVEF owing to cardiac reserve. On the contrary, LV strain derived from 2D-STE may provide more information of both the segmental and global myocardial contractile function. Strain describes deformation of the myocardium during each cardiac cycle in the longitudinal, circumferential, and radial planes. Based on evidence over last decade, LV-GLS has become a feasible alternative to LVEF in evaluating myocardial function and providing additional prognostic information [17], especially in monitoring chemotherapy-induced myocardial toxicity and improving patient management [17–19].

We measured LV-GLS and LV-GCS in this study and found that LV-GLS appeared more sensitive to LV-GCS in demonstrating early myocardial impairment induced by anthracyclines. LV-GLS in the anthracycline group began to decrease as early as cycle-2 of chemotherapy, and more significantly decline was noticed at cycle-4, which is consistent with the known dose-dependent myocardial toxicity of anthracyclines [20]. Patients undertook trastuzumab presented decreased LV-GLS and LV-GCS only at cycle-4, suggesting that early myocardial deformation may not change in a time-frame pattern in response to this type of agent. Notably, when compared the incidence of a meaningful decline of LV strain (> 15% of baseline), patients in group A was more likely to have myocardial impairment at cycle-2 than group B (*P* < 0.05), which consist with previous studies that myocardial deterioration derived from anthracycline may occur earlier than that from trastuzumab [7]. However, the decline rate was found similar at cycle-4 between the two groups, suggesting that the myocardial dysfunction may exaggerate at cycle-4 in trastuzumab group. In summary, 2D-STE is a profound in monitoring subclinical LV impairment and early detecting cardiotoxicity during follow-up of patients with chemotherapy, as recommended by the American Society of Echocardiography and the European Association of Cardiovascular Imaging guidelines [3, 21, 22]. LV-GLS is a more sensitive parameter than LV-GCS in detecting CTRCD. In addition, we found for the first time to our knowledge that 2D-STE may dynamically identify the temporal variation of myocardial impairment caused by different chemotherapy strategy, which may benefit the risk stratification and improve the management of cardiovascular complications.

Furthermore, similar to GLS, all MW parameters had gradual temporal changes in patients with anthracycline, but only GCW in patients with trastuzumab. The increase in GWW and decrease in other MW parameters over time in the anthracycline group probably result from the cumulative dose-dependent cardiotoxicity of type-I CTRCD [20]. MW related parameters are assumed loading-independent and mainly used to describe the dynamic changes noted in the myocardial strain along with the changes of LV pressure during the cardiac cycle, which may reflect myocardial work and metabolic demand [23]. Although GCW is currently the only time-varying parameter in the trastuzumab group, underlying MW deterioration should be cautious as it may be an early sign of LV myocardial fibrosis [24]. This may also suggest that GCW may be a potential key marker that may help identify the earliest myocardial dysfunction in any type of CTRCD. Therefore, in patients without previous cardiovascular disease, MW has potential power in interpreting myocardial impairment. However, MW is not a routine test and evidence to explain CTRCD is lacking currently. Currently, the routine clinical use of MW is limited because it is mainly obtained from a single supplier, thus, the value of MW in CTRCD has not been fully explored. MW is considered superior to GLS in identifying myocardial dysfunction, however, data from multiple centers are needed to determine its benefit in early detection of CTRCD. There are some limitations in this study. The small sample size and short period of follow-up may reduce the efficacy of the study, and we currently only discussed the global performance of myocardium. The critical *P* value for the mean age of the two groups was 0.07, so larger patient cohorts need to be observed. Moreover, given that E/e′ may reflect the LV filling pressure and is useful for evaluating LV diastolic function, there may also exist temporal changes regarding the subclinical LV diastolic dysfunction in patients with different chemotherapy. Also, we currently lack information on MW in detecting pathological and metabolic damage in both types of CTRCD.

## Conclusion

Two-dimensional STE is a pivotal tool to detect and differentiate two types of CTRCD in breast cancer patients. LV-GLS is a profound echocardiographic biomarker in monitoring temporal changes in type-I CTRCD. It also helps to characterize the worsening pattern of LV contractility in type-II CTRCD, which may be mild at cycle-2 but evident as early as cycle-4. In addition, MW provides more evidence of myocardial impairment, while GCW may be the most sensitive marker. Therefore, 2D-STE may contribute to the risk stratification of different CTRCDs and to improve the clinical management of late cardiovascular events in breast cancer patients.

## Data Availability

The dataset used and analyzed during the current study available from the corresponding author on reasonable request.

## Abbreviations

2D-STE: Two-dimensional speckle tracking echocardiography
CTRCD: Cancer therapeutics–related cardiac dysfunction
LVEF: Left ventricular ejection fraction
GLS: Global longitudinal strain
GCS: Global circumferential strain
GCW: Global constructive work
GWE: Global work efficiency
GWI: Global work index
GWW: Global work waste
MW: Myocardial work
